# Delivery of Full-Length Factor VIII Using a *piggyBac* Transposon Vector to Correct a Mouse Model of Hemophilia A

**DOI:** 10.1371/journal.pone.0104957

**Published:** 2014-08-15

**Authors:** Hideto Matsui, Naoko Fujimoto, Noriko Sasakawa, Yasuhide Ohinata, Midori Shima, Shinya Yamanaka, Mitsuhiko Sugimoto, Akitsu Hotta

**Affiliations:** 1 Department of Regulatory Medicine for Thrombosis, Nara Medical University, Kashihara, Nara, Japan; 2 Department of Reprogramming Science, Center for iPS Cell Research and Application (CiRA), Kyoto University, Sakyo-ku, Kyoto, Japan; 3 iCeMS, Kyoto University, Kyoto, Japan; 4 Life Science Experimental Facility, Department of Biotechnology, Faculty of life and Environmental Sciences, University of Yamanashi, 4-4-37 Takeda, Kofu, Yamanashi, Japan; 5 PRESTO, Japan Science and Technology Agency (JST), Saitama, Japan; 6 Pediatrcs, Nara Medical University, Kashihara, Nara, Japan; 7 Gladstone Institute of Cardiovascular Disease, San Francisco, California, United States of America; Justus-Liebig-University Giessen, Germany

## Abstract

Viral vectors have been used for hemophilia A gene therapy. However, due to its large size, full-length Factor VIII (FVIII) cDNA has not been successfully delivered using conventional viral vectors. Moreover, viral vectors may pose safety risks, e.g., adverse immunological reactions or virus-mediated cytotoxicity. Here, we took advantages of the non-viral vector gene delivery system based on *piggyBac* DNA transposon to transfer the full-length FVIII cDNA, for the purpose of treating hemophilia A. We tested the efficiency of this new vector system in human 293T cells and iPS cells, and confirmed the expression of the full-length FVIII in culture media using activity-sensitive coagulation assays. Hydrodynamic injection of the *piggyBac* vectors into hemophilia A mice temporally treated with an immunosuppressant resulted in stable production of circulating FVIII for over 300 days without development of anti-FVIII antibodies. Furthermore, tail-clip assay revealed significant improvement of blood coagulation time in the treated mice.*piggyBac* transposon vectors can facilitate the long-term expression of therapeutic transgenes *in vitro* and *in vivo*. This novel gene transfer strategy should provide safe and efficient delivery of FVIII.

## Introduction

Hemophilia A is a congenital bleeding disorder caused by a deficiency of procoagulation Factor VIII (FVIII). Repeated intravenous injection of recombinant FVIII protein can prevent bleeding events, but alternative treatments could potentially improve patients' quality of life; therefore, hemophilia A is an attractive target disease for the application of gene therapy. Significant advances in both preclinical animal models and phase I/II human clinical trials have been reported [1].

Most preclinical studies of hemophilia A gene therapy have focused on the use of viral vectors, such as adenovirus [2], [3], adeno-associated virus (AAV) [4]–[6], gammaretrovirus [7], and lentivirus [8]–[12]. However, concerns still remain over the risk of adverse immunological reactions against viral proteins [13] and vector-mediated cytotoxicity [14].

Due to the large size of human FVIII cDNA (7.0 kb in total), it cannot be effectively delivered by most existing viral vectors; this technical issue is one of the biggest obstacles to the gene augmentation approach. Consequently, most gene therapy studies, including those aimed at clinical applications, use truncated FVIII that lacks the non-essential B-domain, so called BDD FVIII. However, the B-domain contains several glycosylation sites and is involved in intracellular interactions that regulate protein quality control and secretion; in particular, this domain is presumed to be involved in the clearance of FVIII from plasma [15]. Whether the B-domain is beneficial for FVIII expression in the context of gene therapy remains to be determined.

Recently, non-viral gene-transfer technologies, such as the site-directed integration approach, have been significantly improved [16], [17]. Although conventional plasmid vector systems are inefficient at stably integrating into the target genome, transposon vectors have emerged as attractive gene-delivery tools because of their ability to stably integrate into the genome and achieve efficient and prolonged transgene expression both *in vitro* and *in vivo*. In fact, the Tc1/*mariner* family member *Sleeping Beauty* has been utilized to deliver BDD FVIII into the hemophilia A mouse model [18].

More recently, the *piggyBac* DNA transposon vector was derived from cabbage looper *Trichoplusia ni*. This vector is active not only in insect cells [19], but also in mammalian cells [20]. In HEK293 cells and human primary T cells, *piggyBac* vectors have higher transposition activity than other widely used transposon vector systems, such as *Sleeping Beauty* or *Tol2* [21], [22]. Importantly, the *piggyBac* transposon has a larger cargo size, and can deliver up to 9.1 kb of foreign sequence without significant loss of integration efficiency [20]. Moreover, a recent paper showed that a 100-kb DNA fragment from the *HPRT* gene locus could be inserted into mouse ES cells using *piggyBac*, although the efficiency was low and selective pressure was required [23]. In addition, other groups have shown that *piggyBac* vectors can deliver a reporter gene, such as *lacZ* or luciferase, into mice via hydrodynamic tail-vein injection [24], suggesting that *piggyBac* vectors could be utilized as a non-viral vector for *in vivo* gene therapy applications.

In this study, we investigated the ability of a *piggyBac* vector to deliver full-length FVIII, both in cultured cells and in a mouse model. Injection of *piggyBac* vector into hemophilia A mice resulted in stable and sustained expression of full-length FVIII and improvement of the bleeding phenotype.

## Materials and Methods

### *piggyBac* vector construction

**Figure 1:**
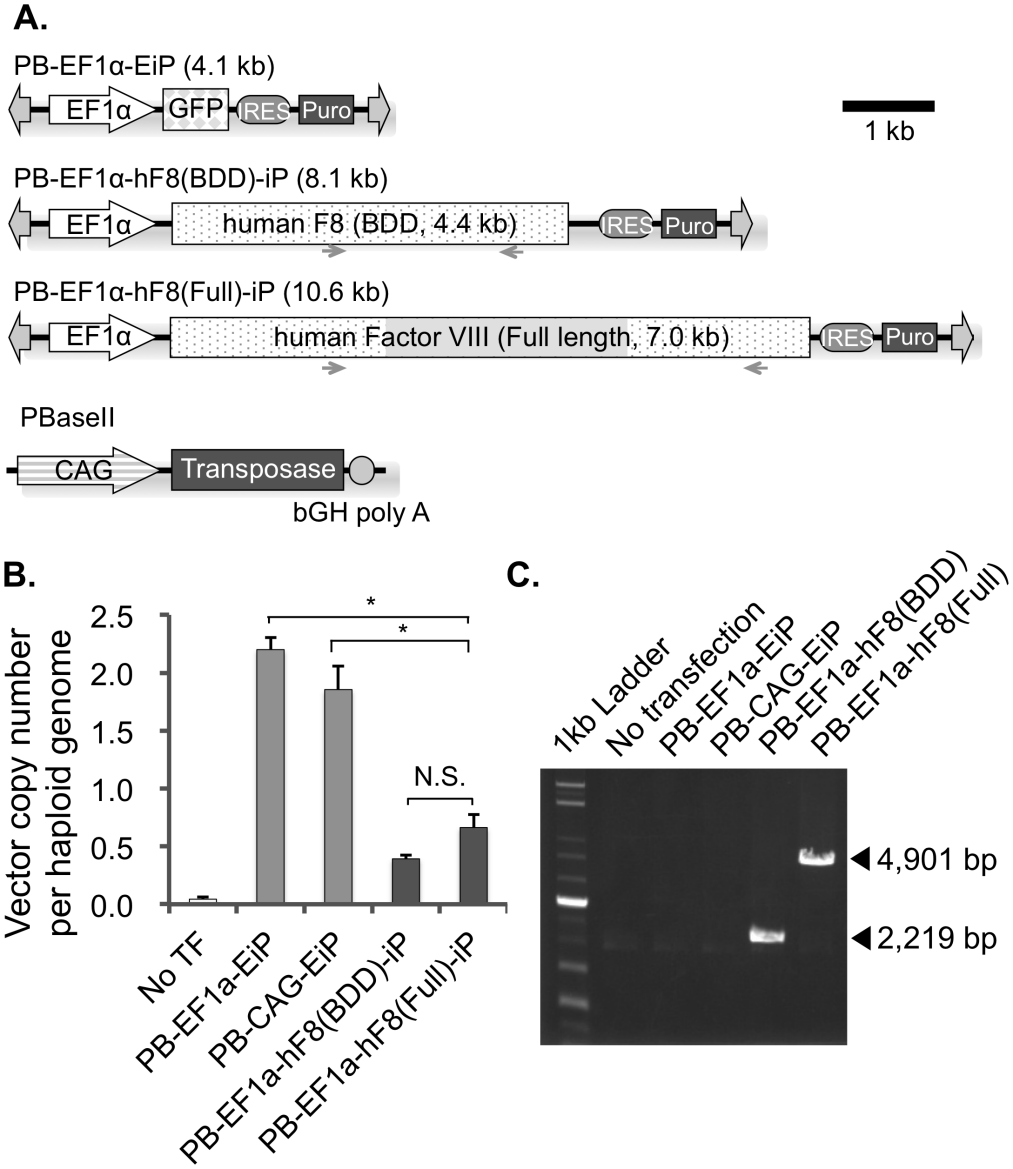
*piggyBac* vectors to express Factor VIII cDNAs. (A) Schematic diagram of *piggyBac* vectors expressing EGFP, B-domain–deleted human FVIII, and full-length human FVIII under the control of the human EF1α promoter. The PBaseII vector expresses *piggyBac* transposase under the control of the CAG promoter. IRES: internal ribosomal entry site. (B) Copy number of genomic *piggyBac* vectors. Indicated *piggyBac* vectors were transfected into 293T cells and selected with puromycin resistance. Approximately three months after transduction, genomic DNAs were extracted, and *piggyBac* vector copy numbers were assessed by real-time PCR using the *piggyBac* 5′ TR primers. The data are normalized to a haploid genome calculated from the copy number of NANOG gene. *: *P*<0.05 by two-sided Student's *t* test (n = 3). N.S.: Not significant. (C) The sizes of inserted FVIII cDNA (2,219 bp for BDD and 4,901 bp for full-length FVIII) were confirmed by genomic PCR using the hF8insertC primers flanking the B-domain (indicated as small arrows in Figure 1A).

A schematic representation of the *piggyBac* transposon vectors used in this study is shown in Figure 1. In brief, to construct the PB-EF1a-EiP vector, the 5′ and 3′ terminal repeat (TR) regions were derived from the PB-MSCII plasmid, based on *Trichoplusia ni* IFP2 *piggyBac* transposon [kind gift from Dr. Knut Woltjen]. Human EF1α promoter was derived from PL-sin-EF1a-EiP [25] as a HindIII–NcoI fragment, and a DNA fragment containing an internal ribosome entry site (IRES) derived from encephalomyocarditis virus and the puromycin resistance gene was obtained by *Nco*I-*Cla*I digestion from the PL-sin-EF1a-EiP plasmid. For construction of PB-EF1a-GWiP, the NcoI (blunted with Klenow)–XbaI fragment of the EF1α promoter and the SacII (blunted by Klenow)–HindIII fragment of the Gateway destination cassette (attR1-Cm^R^-ccdB-attR2) were introduced into the XbaI–HindIII sites of the PB-EF1a-EiP plasmid. For construction of PBaseII, we digested the pCyL-43 plasmid [kind gift from Dr. Knut Woltjen] with SalI and KpnI, blunted it with Klenow, and self-ligated to remove the PGK/puromycin-resistance cassette. The 1.8 kb fragment of HyAcPBase gene [26] with optimized codon usage to human was chemically synthesized (GeneScript). To generate pCAG-HyAcPBase, the gene fragment was cloned into the SacI-NheI site of pCAGGS vector [27].

To construct PB-EF1a-hF8(BDD), BDD FVIII [28] cDNA in the HSQ-MXABS-ReNeo plasmid [29], together with the Adenoviral Type2 promoter and the SV40 poly A site, was digested with NcoI and MfeI and ligated into the NcoI–EcoRI site of PB-EF1a-EiP. Then, the additional SV40 pA site was removed by replacing the HpaI–ClaI SV40 pA-IRES-Puro fragment with the EcoRI (blunted with Klenow)–ClaI fragment of IRES-Puro from PB-EF1a-EiP. Subsequently, the Adenoviral Type2 promoter was removed by replacing the NcoI–MluI fragment with a 1.3-kb PCR fragment amplified from HSQ-MXABS-ReNeo using the following primers (hSQ-FVIII-start-NcoI: 5′-AGCccatggCATGCAAATAGAGCTCTCC-3′; hSQ-FVIII/MluI: 5′-CacgcgtCTTAAAGGTTTCA-3′). To remove the additional ATG start codon, the NcoI site was destroyed by S1 nuclease treatment after NcoI digestion.

Full-length human FVIII cDNA in pENTR 223.1 (pENTR-hF8-full, ID 100066426) was obtained from DNAFORM (Japan), and the entire cDNA sequence was confirmed by Sanger sequencing. Full-length FVIII cDNA in pENTR-hF8-full was subcloned into PB-EF1a-GWiP by Gateway LR recombination.

### *piggyBac* vector transfection *in vitro*

Human embryonic kidney 293T cells were maintained in DMEM media supplemented with 10% fetal bovine serum (FBS), ampicillin, and streptomycin. Lipofectamine 2000 (Life Technologies, CA, USA) was used to transfect 293T cells as described in the manufacturer's protocol. Human iPS cells (201B7 [30]) were maintained in ReproCELL Primate ES Cell media (ReproCELL, Japan) or Knockout-DMEM media (Life Technologies) supplemented with 15% Knockout serum replacement, 2 mM GlutaMAX, 2 mM MEM non-essential amino acids, 0.1 mM 2-mercaptoethanol, and 10 ng/ml recombinant human basic-FGF. Puromycin- and hygromycin-resistant SNL cells (SNL-PH) treated with mitomycin C were used as a feeder layer for human iPS cell cultures. To transfect human iPS cells, we treated the cells with ROCK inhibitor Y-27632 (final 10 µM) for 1 hour, and then dissociated them with CTK solution (0.25% trypsin, 0.1 mg/ml collagenase IV, 1 mM CaCl_2_, and 20% KSR) to remove feeder cells. Human iPS cell colonies were dissociated into single cells using Trypsin–EDTA solution, and then washed twice with Opti-MEM media. We used either the Neon system (Life Technologies) with pulse voltage  = 1200 V, pulse width  = 40 ms, and 1 pulse, or the NEPA21 electroporation system (NepaGene Inc., Japan) with poring pulse voltage  = 125 V and poring pulse width  = 5 ms. Transfection efficiencies of the *piggyBac* vectors with fluorescence reporter were monitored by flow cytometry as previously described [25].

### DNA or RNA extraction and qRT-PCR

For genomic DNA extraction, cell pellets were lysed with 200 µg/ml Proteinase K solution (50 mM Tris-HCl, 20 mM EDTA, 100 mM NaCl, 1%w/v SDS) at 55°C for 3–18 hours, and genomic DNA was extracted by phenol-chloroform-isoamyl extraction method. For RNA, cell samples were lysed using the Trizol reagent (Life Technologies), and total RNA was extracted as described in the manufacturer's protocol. One microgram of RNA was reverse transcribed into cDNA using random primers (9 mer) and Oligo dT (20 mer) with ReverTraAce (Toyobo Inc., Japan).

Real-time quantitative PCR was performed on a StepOnePlus thermal cycler (Life Technologies) with SYBR Select Master Mix (Life Technologies). For genomic copy number analysis, genomic DNA with 3 copies of *piggyBac* vector insertion sites was used as a standard curve, and amount of input genomic DNA was normalized with Ct value by the primers to specifically amplify the NANOG gene region. For cDNA quantification, diluted plasmid DNA (PB-EF1a-EiP) was used to establish a standard curve for the IRES primers, and cDNA input was normalized with the GAPDH primers. Primer sequences are listed in Table S1. Data are presented as means ± standard deviation. The statistical difference was determined by two-sided Student's *t* test, and *P*<0.05 was considered as significant.

### FVIII activity measured by activated partial thromboplastin time (aPTT) assay

To confirm FVIII secretion *in vitro*, supernatant from cell cultures (seeded at 2×10^5^ cells per one well of 12-well plate for 293T cells) was collected 3 days after medium change, centrifuged at 11,000×*g* for 5 minutes to remove cell debris, and then stored at −80°C until being subjected to the aPTT assay as previously reported [10]–[12]. In brief, samples were diluted 10-fold by Owren's Veronal buffer (Sysmex Corporation, Japan) and mixed with FVIII-deficient plasma and aPTT reagent (Sysmex Corporation, Japan). After 3 minutes of incubation at 37°C, the coagulation reaction was activated by addition of 0.02 M CaCl_2_ solution. Coagulation time was measured using a STart4 hemostasis analyzer (Roche Diagnostics). Recombinant human FVIII (Kogenate-FS, Bayer HealthCare Pharmaceuticals, NJ, USA) was used to prepare the standard curve.

### FVIII gene transfer studies in hemophilia A mice

All animal procedures were reviewed and approved by the Nara Medical University Animal Care Committee (Permit Number: 10728). Hemophilia A knockout mice, in which the *FVIII* gene is disrupted by insertion of the *neomycin* gene into Exon 16, were purchased from the Jackson Laboratory (stock number 004424) and back-crossed with C57BL/6 mice. Mice were used at 6–8 weeks of age. Plasmid DNA was diluted in a 10% body-weight volume of lactated Ringer solution and injected via the tail vein within 7 seconds [31]. Because human FVIII is inherently immunogenic in hemophilia A mice, some of the hemophilia A mice also received intraperitoneal injection of cyclophosphamide (20 mg/kg per injection) on the day of vector injection, and then biweekly for 4 weeks. Mouse blood was first collected 1 week after injection from the saphenous vein under isofluorane/oxygen anesthesia, using 10% buffered citrate as an anticoagulant. To generate platelet-poor plasma, collected blood samples were centrifuged at 11,000×*g* for 5 minutes and stored at −80°C prior to testing.

### Chromogenic assay for FVIII activity, FVIII antigen ELISA, and detection of anti-FVIII antibodies

A chromogenic assay was used to measure plasma FVIII functional activity (FVIII: C) as previously described [10]–[12]. Normal pooled human plasma was used to establish the FVIII standard curve; the sensitivity of this assay is 10 mU/mL. In some mouse plasma samples, FVIII antigen (FVIII:Ag) was determined by ELISA (Affinity Biologicals Inc., Ancaster, ON, Canada). Anti-FVIII inhibitory antibodies were measured by the Bethesda assay as previously described [11].

### Tail-clip challenge test

At the termination of the experiments, phenotypic correction was tested in both untreated and vector-treated hemophilia A mice, as previously reported [12]. In brief, mice were anesthetized with isoflurane, and their tails were clipped off at a position near the end of the tail where the cross-sectional diameter was 2.0 mm. The mice were then observed to determine the bleeding time.

### Immunohistochemical analysis of FVIII in multiple organs

Immunohistochemical analysis of FVIII expression in both untreated and vector-treated hemophilia A mice were performed as previously reported [11], [12] with minor modifications. In brief, after tail-clip challenge tests, the mice were sacrificed, multiple organs liver, spleen, kidney, heart, and lung) were harvested, fixed with 10% formalin, embedded in paraffin and sectioned for staining. To assess transgene expression in tissues, specimens were stained with Cy3-labeled human anti-FVIII IgG [32] using the FluoroLink Ab labeling kit (Amersham Biosciences, Buckinghamshire, UK). Staining images were captured with FV300 confocal laser scanning microscope (Olympus Co., Tokyo, Japan).

## Results

### Stable expression of FVIII by *piggyBac* vector in multiple cell types

In order to achieve high-level transgene expression, we first optimized the internal promoter of our *piggyBac* vector. We constructed EGFP-expressing *piggyBac* vectors containing various promoters including those of PGK, CAG, and Elongation factor 1α (EF1α or EEF1A1). The EF1α promoter was the strongest among those we tested (Figure S1). Next, we optimized the amount of the *piggyBac* vector DNA and transposase-expressing plasmid. In agreement with previous reports [33], we found that a higher amount of *piggyBac* vector and a lower amount of transposase (400 µg/µl and 50 µg/µl, respectively) were optimal for transduction of our *piggyBac* vectors (Figure S2). We also tested a hyperactive version of the *piggyBac* transposase [24], [26], but observed no significant improvement in transduction efficiency relative to the wild-type transposase (data not shown). To examine the effect of vector size on transduction efficiency, we constructed mCherry-expressing *piggyBac* vectors containing cDNAs of various sizes. Although the transduction efficiencies were reciprocally correlated with the vector sizes, the full-length FVIII cDNA (10.6 kb) could be transduced as efficiently as the BDD FVIII cDNA (Figure S3). In fact, when we transfected *piggyBac* vectors expressing EGFP, BDD FVIII, or full-length FVIII (Figure 1A), we observed that full-length FVIII vector could integrate into host chromosomes as efficiently as BDD FVIII vector (Figure 1B).

**Figure 2:**
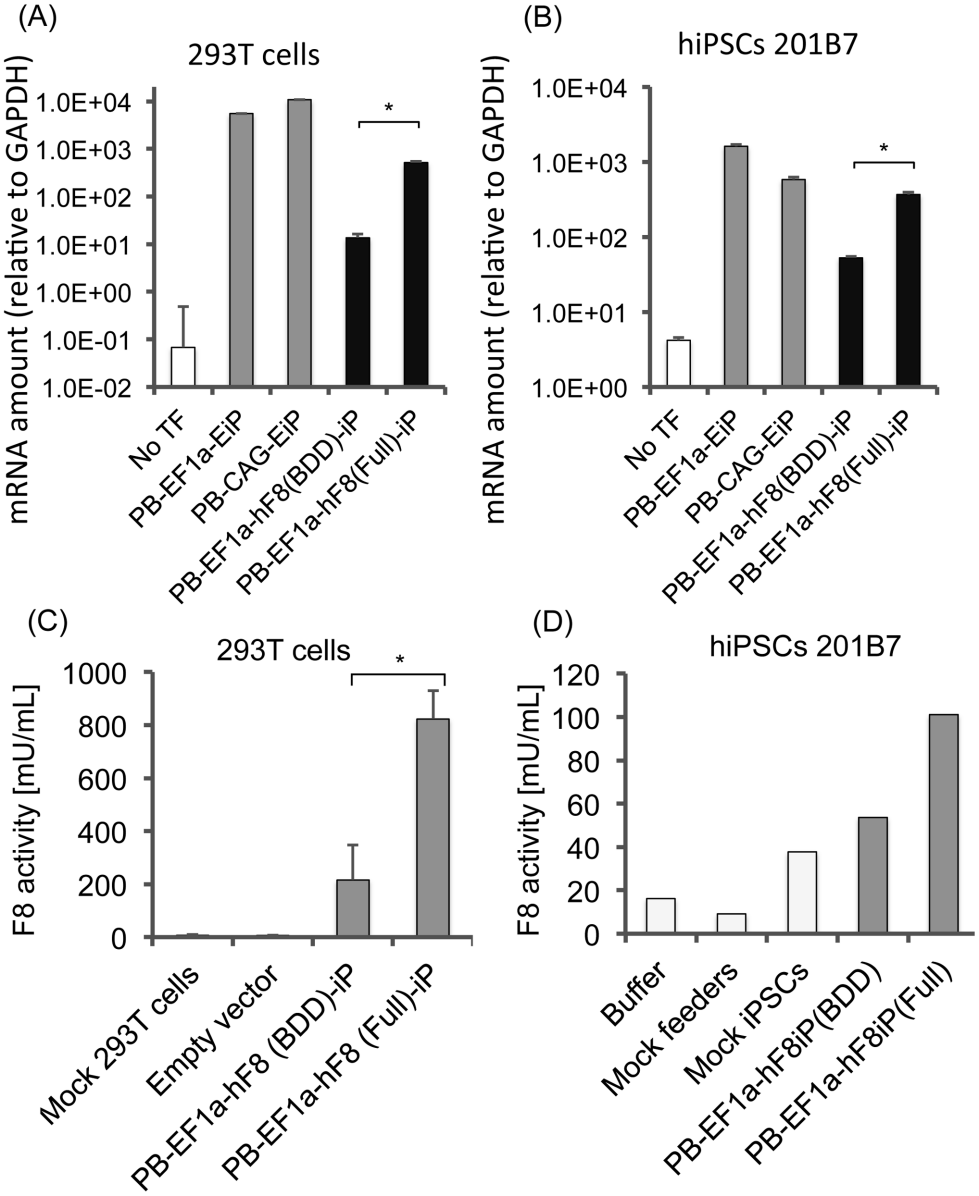
Full-length FVIII can be expressed at levels as high as BDD FVIII. (A, B) Total mRNAs were extracted from 293T cells (A) or human iPS cells (B), and the level of expression was assessed by quantitative RT-PCR. Expression values were normalized to the level of *GAPDH* mRNA. (C, D) Secretion of functional FVIII measured by aPTT assay. Culture supernatants of transfected cells were harvested 3 days after medium change and subjected to aPTT assay to measure the coagulation activity of secreted FVIII protein in 293T cells (C) or human iPS cells (D). Recombinant FVIII product was used to generate a standard curve. Normal FVIII activity (100%) represents 1 U/ml ( = 1000 mU/ml). *: *P*<0.05 by two-sided Student's *t* test (n = 3).

Next, to evaluate the expression of FVIII cDNAs by *piggyBac* vectors, we transfected these vectors into 293T cells and human iPS cells (clone 201B7); the former cell type is easy to transfect, whereas the latter type retains a normal karyotype while retaining unlimited self-renewal capacity and pluripotency [34]. We then extracted total RNA from these cells and quantitated transcription levels from *piggyBac* vectors by qRT-PCR using IRES primers. Even though the transcription levels of the FVIII vectors were lower than those of the of EGFP control vectors, comparable levels of transcription were detected from BDD and full-length FVIII vectors (Figure 2A,B).

To assess the level of secreted and functional FVIII protein, we measured the FVIII coagulation activity in the culture supernatant by activated partial thromboplastin time (aPTT) assay. We observed higher coagulation activity with full-length FVIII than BDD FVIII (Figure 2C,D). We also observed lower secretion of FVIII protein in undifferentiated iPS cells than in 293T cells, partly due to the lower transduction efficiency of iPS cells. The undifferentiated state of iPS cells may be associated with immaturity of the secretory pathways involved in FVIII production.

### Successful long-term phenotypic correction of hemophilia A mice by hydrodynamic injection with *piggyBac* vectors

**Figure 3:**
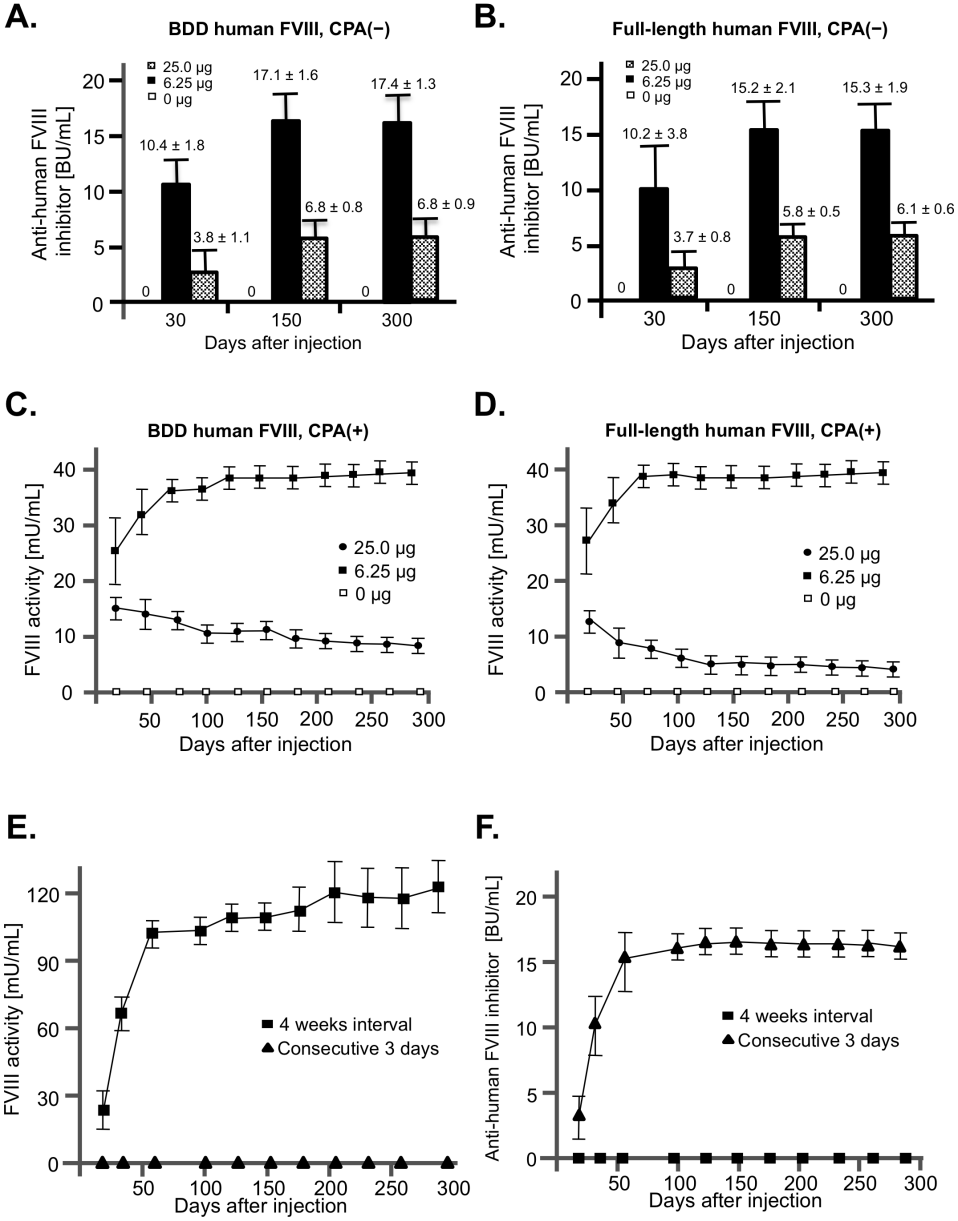
Long-term and stable expression of FVIII in hemophilia A mice. (A, B) *piggyBac* vector (25 µg of DNA) expressing either B-domain–deleted (A) or full-length FVIII (B) was introduced into hemophilia A mice (n = 5 each) by hydrodynamic injection without cyclophosphamide treatments. Amount of PBaseII *piggyBac* transposase (0 µg, 6.25 µg, or 25 µg of DNA) is indicated by symbols. Levels of anti-human FVIII inhibitors in mouse plasma were measured by Bethesda assay. (C, D) *piggyBac* vector (25 µg of DNA) expressing either B-domain-deleted (C) or full-length FVIII (D) was introduced into hemophilia A mice (n = 7 each) by hydrodynamic injection with cyclophosphamide treatments. Amount of PBaseII *piggyBac* transposase (0 µg, 6.25 µg or 25 µg of DNA) is indicated by symbols. Levels of FVIII activity in mouse plasma were measured by chromogenic assay. (E, F) Multiple injections boosted the level of FVIII in hemophilia A mice. PB-EF1α-hFVIII(Full)-iP (25 µg) and PBaseII (6.25 µg) vectors were hydrodynamically injected into hemophilia A mice (n = 7 each) three times at intervals of 24 hours or 4 weeks. (E) Level of FVIII activity in mouse plasma was measured by chromogenic assay. (F) Level of anti-human FVIII inhibitor was measured by Bethesda assay.

As a proof-of-concept that *piggyBac* vectors are applicable to gene therapy applications, we injected FVIII-expressing *piggyBac* vectors into hemophilia A model mice. Because FVIII is mainly produced in the liver, we targeted the liver by hydrodynamic injection via the tail vein. Human FVIII is inherently immunogenic in hemophilia A mice; consequently, all recipient mice that did not receive immunosuppresive treatment developed inhibitory anti–human FVIII antibodies, and there was no difference in the titer levels of FVIII antibodies between BDD and full-length FVIII vectors (Figure 3A, B). Therefore, under transient immunosuppression with cyclophosphamide, we injected either BDD or full-length FVIII *piggyBac* vectors (25 µg) with various amounts of transposase (0 µg, 6.25 µg, or 25 µg). As expected from the *in vitro* results (Supplemental Figure 2), we observed higher FVIII level in plasma of animals that received lower amounts of transposase. The levels of FVIII in plasma were 25–40 mU/ml (2.5–4.0% of normal level) in all hemophilia A mice injected with 6.25 µg of transposase (Figure 3C, D). Furthermore, we observed that the activity levels of full-length FVIII (Figure 3D) were comparable to those of BDD FVIII (Figure 3C), despite the size difference between the two proteins. In addition, the levels of FVIII antigen determined by human-specific FVIII ELISA corresponded with FVIII activity, as revealed by chromogenic assay in the same plasma samples (data not shown). The plasma levels of FVIII were stably maintained for more than 300 days.

In an attempt to boost the production of FVIII, we tested the effect of multiple injections of *piggyBac* vector. Surprisingly, when we injected the vector at 24-hour intervals, no FVIII production was observed (Figure 3E). Rather, we observed the production of FVIII inhibitors in response to the injections (Figure 3F). On the other hand, multiple vector injections at 4-week intervals boosted the levels of FVIII expression in hemophilia A mice without causing development of FVIII antibody (Figure 3E, F).

**Figure 4:**
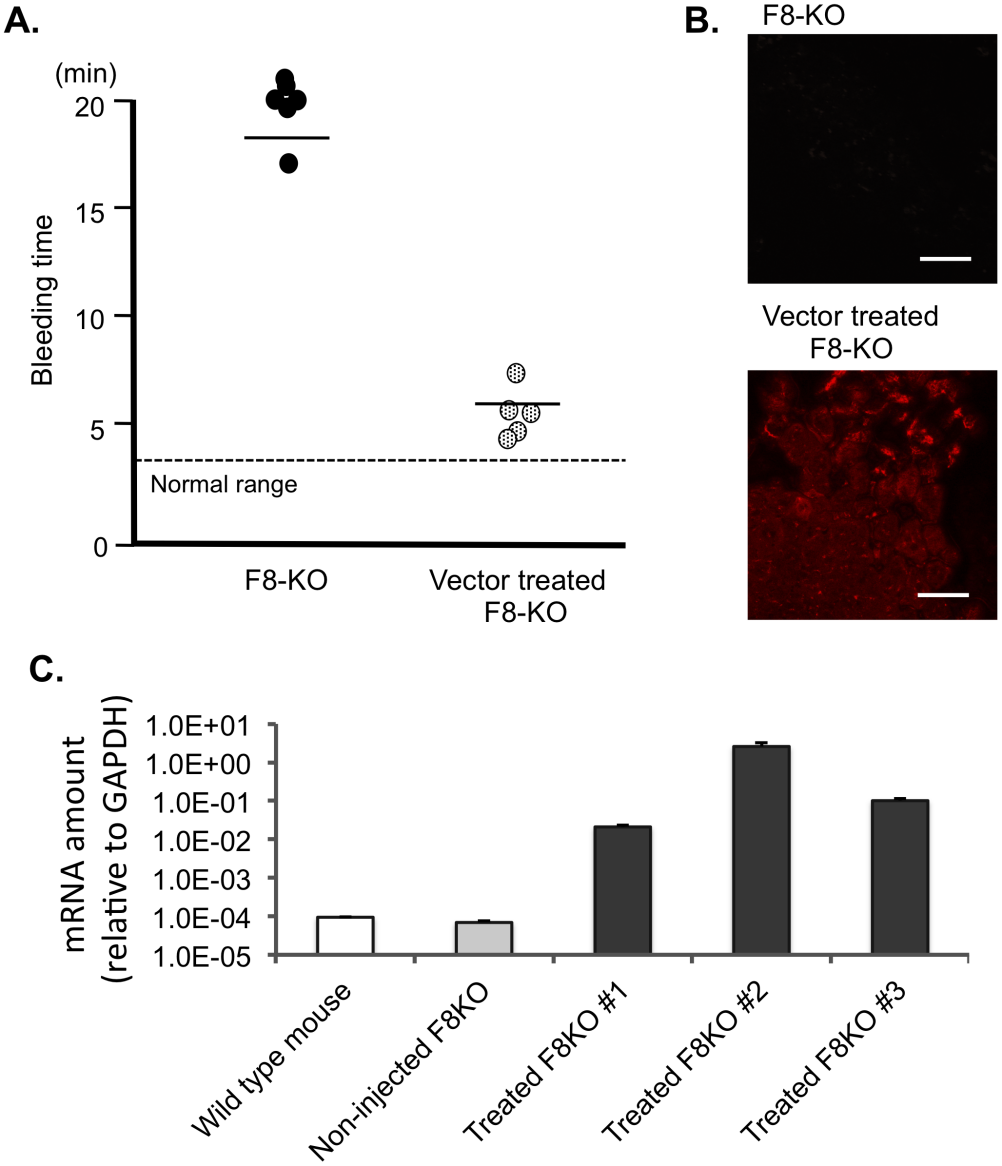
Phenotypic correction of hemophilia A mice by injection of *piggyBac* vectors. (A) Bleeding time of both hemophilia A mice (n = 6) and hemophilia A mice treated with *piggyBac* vector expressing full-length Factor VIII (n = 5) assessed by tail-clip assay. (B) Immunohistochemical analysis of liver tissue from non-treated hemophilia A mice and hemophilia A mice treated with *piggyBac* vector expressing full-length FVIII. Scale bar represents 50 µm (x400: original magnification). (C) Total RNA were extracted from the mouse liver treated with *piggyBac* vectors with 4 weeks interval, and quantified the level of transgene mRNA by qRT-PCR using human FVIII light-chain primers. Expression values were normalized to the level of *GAPDH* mRNA.

Next, to determine whether the *piggyBac* vector injection corrected the bleeding diathesis phenotype, we subjected the mice to tail-clip challenge. The tails of treated and untreated hemophilia A mice from Figure 3D were transected at a position corresponding to a specific diameter, and then bleeding times were monitored. Whereas untreated hemophilia A mice (n = 6) tended to lack stable clot formations, all hemophilia A mice treated with *piggyBac* vector (n = 5) formed a stable clot after tail clipping (Mean bleeding time; Untreated hemophilia A mice: 18 min 24 sec, Vector-treated hemophilia A mice: 6 min 13 sec, respectively) as shown in Figure 4A.

Finally, the mice were sacrificed, and multiple recovered organs were examined for FVIII expression by immunohistochemical staining. Among the organs we examined, only liver expressed detectable level of human FVIII protein (Figure 4B). We also confirmed the expression of human FVIII transcripts in homogenized liver by qRT-PCR (Figure 4C). Thus, even though FVIII expression was controlled by the ubiquitously active EF1α promoter, the expression pattern achieved by the hydrodynamic-injection method is largely restricted in liver.

Overall, these data indicate that *piggyBac* vectors can mediate efficient and sustained expression of the full-length FVIII gene, and thereby rescue the bleeding phenotype of hemophilia A mice.

## Discussion

The continuous improvement of gene-transfer vectors has broad implications for genetic studies and gene-therapy applications. In the gene-therapy field, host immunoresponse to viruses pose a major challenge for virus-mediated gene transfer, especially in the case of adenoviral or AAV vectors [2], [3], [6], [14], [35], [36]. In addition, some hemophilia patients still suffer from AIDS due to the contamination of HIV in clotting factor concentrates that were used for the treatment. To avoid the risk of recombination between viral vectors and wild-type viruses, the use of relevant lentiviral vectors [8]-[11] should be avoided in patients who have a history of infection with HIV. Another limitation of viral vectors relates to the costly and cumbersome manufacturing process, which poses significant regulatory hurdles. Therefore, there is a need to develop safe and efficient non-viral gene delivery approaches that diminish the immunoresponse against viral components, overcome some of the manufacturing and regulatory hurdles, and allow for stable expression of the therapeutic gene(s).

Currently available non-viral plasmids integrate inefficiently, and gene expression from these plasmids typically declines within days after transfection. To overcome these issues, we have attempted to establish non-viral gene delivery approaches that take advantage of the latest transposon technology [19], [20]. Because *piggyBac* transposons integrate into the target-cell genome, they have the potential for long-term expression of therapeutic genes.

Here, we performed hydrodynamic injection of *piggyBac* vectors into hemophilia A mice, resulting in stable and long-term expression of BDD and full-length FVIII in plasma. Furthermore, the bleeding phenotype of hemophilia A mice was almost completely abolished after transduction with *piggyBac* vectors. Although hydrodynamic transfection is not readily applicable to a clinical setting, our study demonstrates proof-of-principle that *piggyBac* vectors could be used for hemophilia gene therapy, because these vectors enjoy several advantages relative to previously developed viral vector–mediated gene transfer approaches.

First, we took advantage of the large cargo size of *piggyBac* vectors to deliver full-length FVIII cDNA. Despite the difference in the sizes of the two vectors (8.1 kb and 10.6 kb), we observed comparable transduction efficiencies between BDD and full-length FVIII vectors. The B-domain of FVIII is not essential for coagulation activity; however, previous reports have suggested that it plays a role in protein secretion and quality control [15]. Such a function of the B-domain may influence the overall FVIII activity under some circumstances. Our *piggyBac* vector should provide a powerful platform for investigating the effect and biochemical properties of the B-domain and its variants.

Second, *piggyBac* vector–mediated gene transfer offers a simple and cost-effective gene delivery system, because gene transfer can be achieved simply by co-transfection of vector DNA and transposase. This is in contrast to viral vector preparation, which requires a packaging cell line, several helper plasmids, concentration, and purification. The simplicity of the production procedure and lower cost should facilitate the manufacture of *piggyBac* vectors for clinical applications.

On the other hand, several problems must be solved before transposon-based vectors can be used in therapeutic applications. Similar to gammaretroviruses or lentiviruses, *piggyBac* vector preferentially integrate their DNA sequences into host chromosomes near transcriptional start sites [21]; therefore, insertional mutagenesis and genotoxicity remain significant concerns. Indeed, insertion of non–self-inactivating retroviral vectors has resulted in the activation of oncogenes in human patients [37], [38]. More recently designed self-inactivating retroviral vectors are less likely to activate surrounding genes via their LTR promoters; however, the possibility of *trans*-activation by internal enhancers/promoters must still be taken into account [39]. The degree of genotoxicity mediated by our *piggyBac* vector remains to be determined.

Hemophilia A patients require a continuous supply of active FVIII protein; therefore, for gene-therapy applications, it is preferable to achieve stable and sustained expression of FVIII in order to achieve consistent therapeutic outcomes and reduce the risk of developing inhibitory antibodies. Our approach to *in vivo* delivery by hydrodynamic injection cannot control the integration sites or targeted cells. However, the *ex vivo* gene therapy approach using tissue stem cells, BOECs [11], [12], or patient-derived iPS cells enables us to pre-determine the integration sites of the vectors. The *ex vivo* approach allows us to select for cell clones that effectively secrete active FVIII and the vector has integrated into a “safe harbor” in the human genome, however, suitable cell type(s) for *ex vivo* targeting need to be determined.

Another limitation of the *piggyBac* vector is its delivery method. Hydrodynamic injection is a well-established and efficient delivery system in small animals [31], [40], but not readily applicable to a human clinical setting for hemophilia patients as the method carries a risk of liver damage and bleeding. This warrants the development of alternative *in vivo* DNA delivery approaches. More controlled (smaller buffer volume, but with similar hydrodynamic pressure for purposes of gene transfer) and local hydrodynamic injection methods using catheter are preferred [41], [42]. Transposon and transposase constructs can be delivered to target cells by chemical transfection with carrier molecules that facilitate their entry. Physical transfection methods such as electroporation have been used for *ex vivo* gene delivery of transposon/transposase constructs, resulting in long-term and efficient transgene expression. Furthermore, recent advances in tissue-specific DNA delivery methods based on nanotechnology [43] or chemical modifications may accelerate the delivery of *piggyBac* vectors *in vivo*.

In conclusion, we successfully achieved long-term therapeutic expression of full-length FVIII gene *in vitro* and *in vivo* by non-viral *piggyBac* vectors. The present study is the first report demonstrating that *piggyBac* vectors can mediate sustained FVIII expression *in vivo*, and provides evidence that *piggyBac* is a promising tool for various *in vivo* gene-transfer applications. We believe that the present study will encourage the development of *piggyBac*-based vectors, and contribute to future *in vivo* gene and cell therapy for genetic diseases including hemophilia.

## Supporting Information

Figure S1 **Optimization of internal promoters.** *piggyBac* vectors expressing an EGFP-IRES-puro cassette under the control of various promoters (no internal promoter, PGK, CAG, or EF1α) were transfected into human iPS cells (A). The GFP fluorescence intensities from three samples were assessed by flow cytometry, and error bars represent the standard deviation (B).
(EPS)

Figure S2 **DNA ratio of** ***piggyBac*** **vector and transposase.** Either wild-type *piggyBac* transposase (A) or hyperactive *piggyBac* transposase (B) was co-transfected with various amount of *piggyBac* vector (PB-EF1α-EiP) into 293T cells. Transduction efficiencies were measured by flow cytometry 14 days after transfection. Transduction efficiencies for each condition are indicated by percentage (%) and grayscale (low transduction efficiency: light gray, high transduction efficiency: dark gray). N.E.: Not examined.
(TIFF)

Figure S3 **Size of** ***piggyBac*** **vectors and transduction efficiency.** Twenty-eight *piggyBac* vectors of various sizes (PB-EF1a-cDNA-IRES-mCherry) were transfected into 293T cells. Percentages of mCherry-positive cells were measured by flow cytometry, and each vector is plotted on the graph as a gray diamond. Transduction efficiency (*E* [%]) of the *piggyBac* vectors and their vector sizes (*S* [bp]) were correlated with the approximate power function (dotted line, *E* = 3,380,333*S* ^−1.401^); the coefficient of determination (R^2^) was 0.717.
(EPS)

Table S1 **List of primers used in this study.**
(TIFF)
