# Supplementary figures and images for: Delivery of Full-Length Factor VIII Using a piggyBac Transposon Vector to Correct a Mouse Model of Hemophilia A
Source: PLoS One. 2014 Aug 15;9(8):e104957. doi: 10.1371/journal.pone.0104957 (PMC4134236; doi:10.1371/journal.pone.0104957)

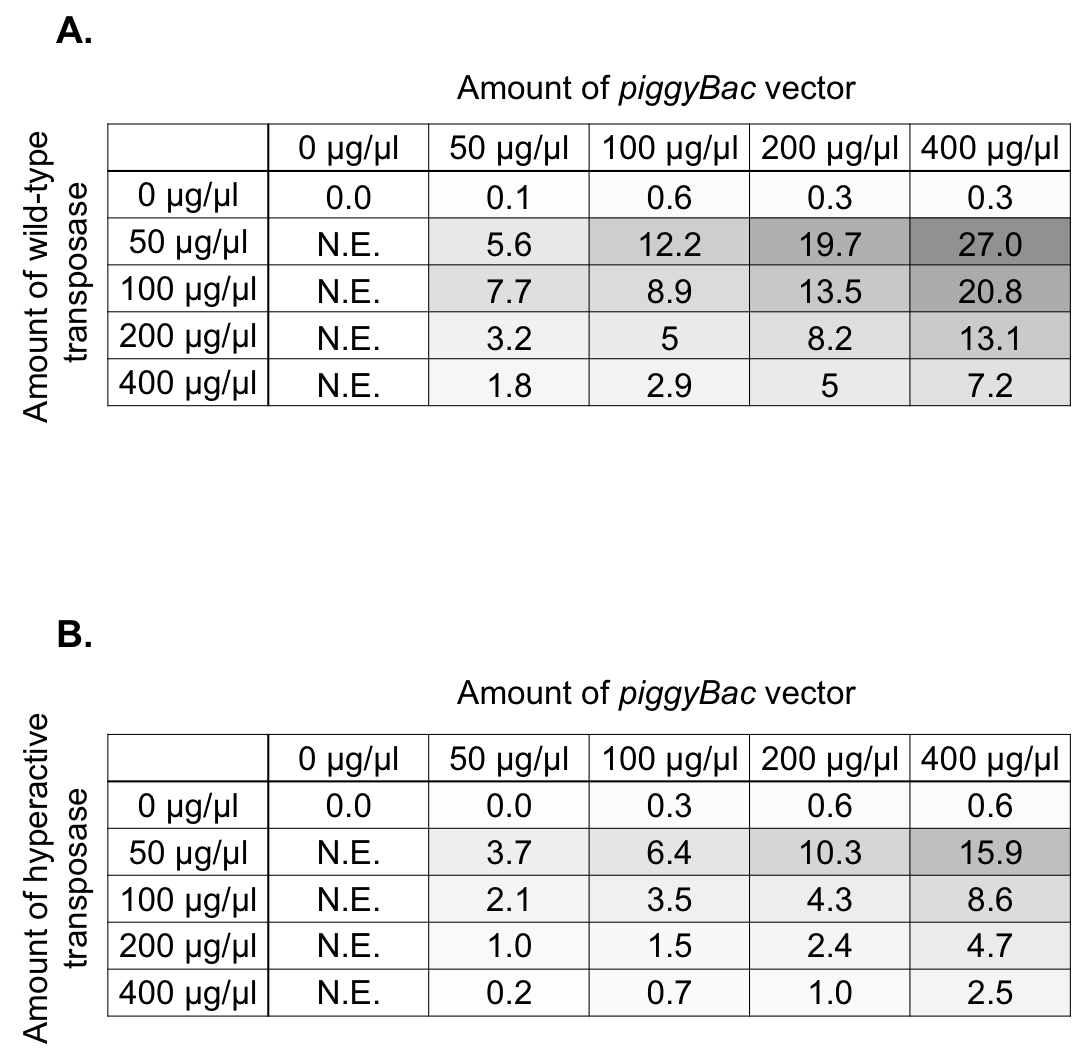

Supplement: Figure S2 — DNA ratio of piggyBac vector and transposase. Either wild-type piggyBac transposase (A) or hyperactive piggyBac transposase (B) was co-transfected with various amount of piggyBac vector (PB-EF1α-EiP) into 293T cells. Transduction efficiencies were measured by flow cytometry 14 days after transfection. Transduction efficiencies for each condition are indicated by percentage (%) and grayscale (low transduction efficiency: light gray, high transduction efficiency: dark gray). N.E.: Not examined. (TIFF) [file pone.0104957.s002.tiff]
